# Supplementary material for: The interactome of intact mitochondria by cross-linking mass spectrometry provides evidence for coexisting respiratory supercomplexes
Source: Mol Cell Proteomics. 2017 Dec 8;17(2):216–32. doi: 10.1074/mcp.RA117.000470 (PMC5795388; doi:10.1074/mcp.RA117.000470)
Supplement: Supplemental Data [file supp_RA117.000470_133922_0_supp_23978_fzffwf.zip › spectra_annotation/mito_spectra_annotation/153-1-1-1-2-1.pdf]

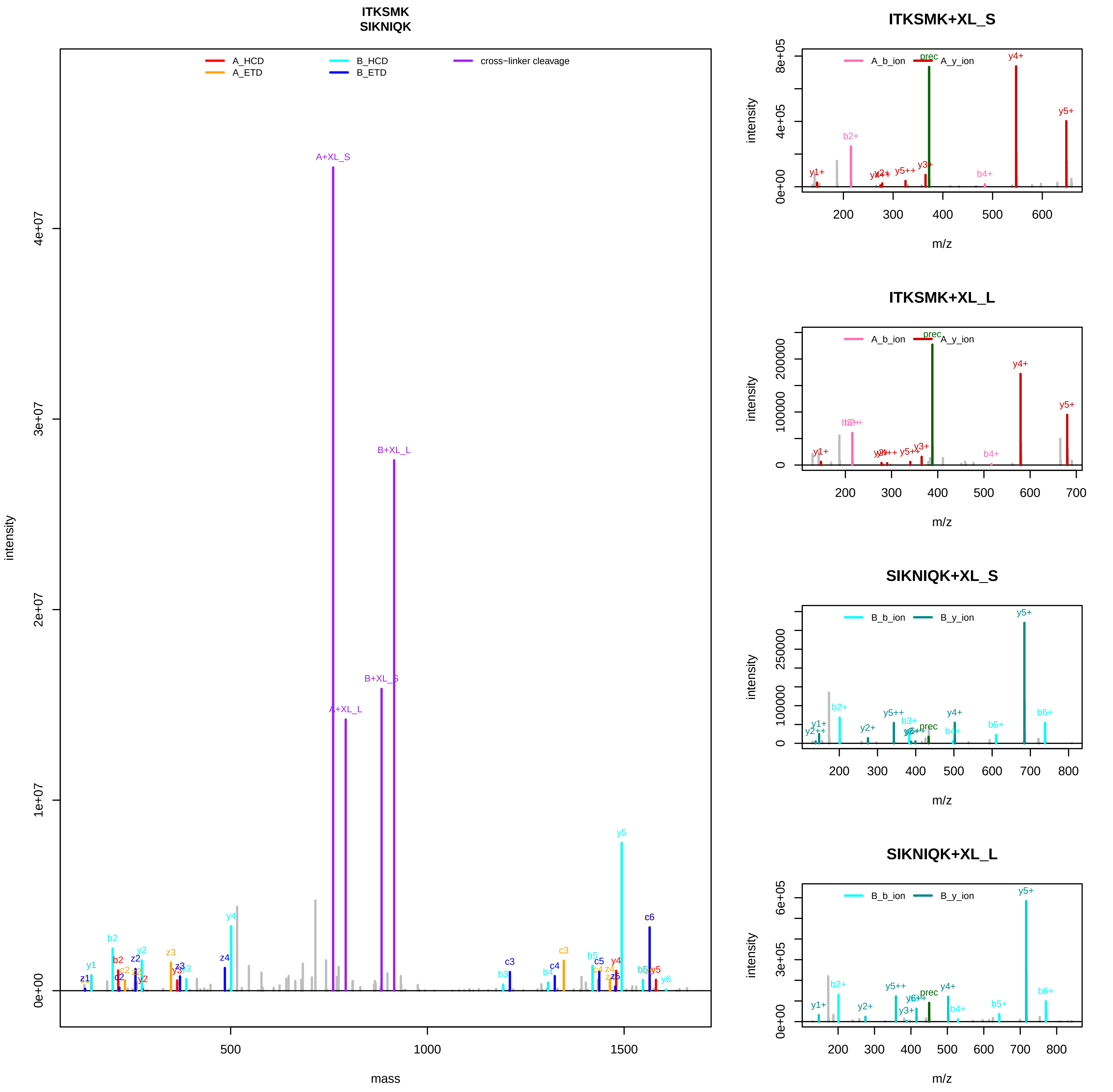
ITKSMK
SIKNIQK
A_HCD
A_ETD
B_HCD
B_ETD
cross−linker cleavage
A+XL_S
B+XL_L
B+XL_S
A+XL_L
intensity
mass
0e+00
1e+07
2e+07
3e+07
4e+07
500
1000
1500
y1
z1
b2
c2
z2
y2
z3
y3
b3
z4
y4
c3
b4
c4
c5
b5
y5
c6
y6
ITKSMK+XL_S
ITKSMK+XL_L
SIKNIQK+XL_S
SIKNIQK+XL_L
A_b_ion
A_y_ion
B_b_ion
B_y_ion
prec
y1+
b2+
y2+
y3+
y4+
y5+
y5++
b4+
b3+
b5+
b6+
y2++
y6++
m/z
8e+05
4e+05
6e+05
3e+05
250000
200000
150000
100000
50000
0
200
300
400
500
600
700
800
